# A Novel Branched Chain Amino Acids Responsive Transcriptional Regulator, BCARR, Negatively Acts on the Proteolytic System in *Lactobacillus helveticus*

**DOI:** 10.1371/journal.pone.0075976

**Published:** 2013-10-11

**Authors:** Taketo Wakai, Naoyuki Yamamoto

**Affiliations:** 1 Microbiology and Fermentation Laboratory, Calpis Co., Ltd., Sagamihara-shi, Kanagawa, Japan; 2 Research and Development Planning Department, Calpis Co., Ltd., Sagamihara-shi, Kanagawa, Japan; Beijing Institute of Microbiology and Epidemiology, China

## Abstract

Transcriptional negative regulation of the proteolytic system of *Lactobacillus helveticus* CM4 in response to amino acids seems to be very important for the control of antihypertensive peptide production; however, it remains poorly understood. A 26-kDa protein with N-terminal cystathionine β-synthase domains (CBS domain protein), which seems to be involved in the regulatory system, was purified by using a DNA-sepharose bound 300-bp DNA fragment corresponding to the upstream regions of the six proteolytic genes that are down-regulated by amino acids. The CBS domain protein bound to a DNA fragment corresponding to the region upstream of the *pepV* gene in response to branched chain amino acids (BCAAs). The expression of the *pepV* gene in *Escherichia coli* grown in BCAA-enriched medium was repressed when the CBS domain protein was co-expressed. These results reveal that the CBS domain protein acts as a novel type of BCAA-responsive transcriptional regulator (BCARR) in *L. helveticus*. From comparative analysis of the promoter regions of the six proteolysis genes, a palindromic AT-rich motif, 5′-AAAAANNCTWTTATT-3′, was predicted as the consensus DNA motif for the BCARR protein binding. Footprint analysis using the *pepV* promotor region and gel shift analyses with the corresponding short DNA fragments strongly suggested that the BCARR protein binds adjacent to the *pepV* promoter region and affects the transcription level of the *pepV* gene in the presence of BCAAs. Homology search analysis of the C-terminal region of the BCARR protein suggested the existence of a unique βαββαβ fold structure that has been reported in a variety of ACT (aspartate kinase-chorismate mutase-tyrA) domain proteins for sensing amino acids. These results also suggest that the sensing of BCAAs by the ACT domain might promote the binding of the BCARR to DNA sequences upstream of proteolysis genes, which affects the gene expression of the proteolytic system in *L. helveticus.*

## Introduction

Among *Lactobacillus* species, *Lactobacillus helveticus* is widely used as a starter culture in the manufacture of a variety of fermented dairy products [1], such as yogurt and Swiss cheese [2]. *L. helveticus* is a Gram-positive, nonspore-forming microaerophilic rod [3] that can grow rapidly in milk because of its high proteolytic activity, which allows it to utilize peptides and amino acids released from the hydrolysis of milk proteins [4]. The use of *L. helveticus* in the production of dairy products has received increased attention because of the organism’s ability to generate antihypertensive peptides from casein during the milk fermentation process [5]. The antihypertensive effect was shown to be specific to *L. helveticus* fermented milk in a study using spontaneously hypertensive rats [5]. Key active components of the antihypertensive effect of *L. helveticus* fermented milk are considered to be Val-Pro-Pro (VPP) and Ile-Pro-Pro (IPP) [6], [7], which inhibit angiotensin I-converting enzyme (kininase II; EC 3.4.15.1) [8]. A highly active cell wall-associated proteinase and several intracellular peptidases specific to *L. helveticus* strain CM4 are thought to be responsible for the organism’s ability to release large amounts of these two antihypertensive tripeptides [9]. However, the production of VPP and IPP by *L. helveticus* CM4 is mildly repressed by peptides [10] and amino acids that accumulate in fermented milk as a result of the down-regulation of genes such as *pepO2*, *pepCE* and *pepE*, which most likely encode enzymes involved in the processing of both bioactive peptides [11]. In *Lactococcus lactis*, most of the genes regulated by the CodY protein in response to branched chain amino acids (BCAAs) are involved in the proteolysis system [12]. In *L. lactis* and *Bacillus subtilis*, BCAAs modulate the activity of CodY by increasing the affinity of CodY for its operator sites [13]–[15]. In *B. subtilis*, an additional level of regulation of CodY activity is provided by GTP, an indicator of the energy state of the cell, which stimulates CodY activity independent of BCAAs [16], [17].

In the previous study, the existence of a CodY-like regulatory system controlling expression of the *pepO*, *pepO2*, *pepV*, *pepCE*, *pepT2* and *dppD* genes in response to amino acids was suggested by transcriptome analysis of *L. helveticus* CM4 [11]. However, there are no CodY-like homologs in lactobacilli and no reports of a regulator in lactobacilli to explain the repressive effect on the proteolytic system by amino acids. We predicted that *L. helveticus* CM4 has a novel regulatory protein with affinity for DNA sequences located upstream of these proteolysis genes, and therefore we attempted to purify a regulatory protein with a DNA-affinity resin to identify the regulatory protein. Here we report the characterization and structural features of a novel regulatory protein from *L. helveticus* CM4, and demonstrate its ability to bind to a specific DNA-motif located upstream of genes encoding proteolytic enzymes to control their expression.

## Materials and Methods

### Preparation of DNA-sepharose

Six pairs of primers were designed to amplify the promoter regions (approximately 300 bp each) controlling expression of 6 proteolysis genes (Table 1) known to be down-regulated in the presence of peptides in *L. helveticus* CM4 [11]. To amplify the promoters for the *pepCE* and *pepO* genes, which are not the first genes in each operon, the putative promoter region of the first gene in each operon was used. Six biotynylated PCR fragments, which were generated by PCR with six sets of biotynylated primers and CM4 genomic DNA, were mixed with Streptoavidin-sepharose (Sigma Aldrich) to prepare DNA-sepharose.

**Table 1:** Primers used in this study.

Experiments	Name	Sequence (5′-3′)
Affinity sepharose and EMSA* for 6 genes		
	dppDupF	AACTTGAAGATGAATTTGGC
	dppDupR	CTAGTAATAGATCACTCTGC
	pepVupF	TCCATCCTATCGCTTAAAGG
	pepVupR	TCATCTTTTTTAGCAGCAGC
	pepDupF	GCTACAAACATTGTGTCAGC
	pepDupR	CTTGCTCGTAACTTAAATCG
	pepO2upF	CCGTCAGCATCAACAGTTGC
	pepO2upR	CGCGGATTTTTGCTAAATTC
	pepOupF	CAGCTTCATAGATTTTATGC
	pepOupR	ATATAGCGCTTCTTTTCCTG
	pepC2upF	CCACTGTTCCAGTTTTCATC
	pepC2upR	TTCTTGTCGGCATGGTATCC
EMSA for specific pepV gene fragments		
	pepV_−266/−166_F	TCCATCCTATCGCTTAAAGG
	pepV_−266/−166_R	AGAGCAACCATGCATCGAAA
	pepV_−216/−116_F	GAGTCCTATCAAACTATATA
	pepV_−216/−116_R	ATGTACAGTTGATCTTGAAA
	pepV_−136/+4_F	TTTCAAGATCAACTGTACATTCATGATAGT
	pepV_−136/+4_R	TTCATATATTTCTCTCCCTT
	pepV_−76/+43_F	GTATCTTATTTTTAGTAAGAATCAAACATT
	pepV_−76/+43_R	TCATCTTTTTTAGCAGCAGC
Preparation of GST fusion protein		
	26kF	GGAGGCGAATTCATGCTTATTAAATCTTTAGTC
	26kR	ATTGCAGGGTATTTAACTCGAGCACCACTACAATTC
Preparation of the plasmids for transcriptional analysis		
	pepVexF	GTATTCGGATCCAGTTGCTTCTGCTTTTGC
	pepVexR	AAGGGAATTCCATGGATGATGGTTTAATGG
	26kexF	CGGGATCCGTACTGCTTCAGCAATGATCGC
	26kexR	GTTGAGCATGCCAACAAGGAATTTGCTGTG
Real-Time PCR for transcriptional analysis		
	pepVRTF	AAGGACAATATCCGTTACCC
	pepVRTR	GAACGTAGTGAGGTTCTTCG
	gapRTF	GTTCACGCTACTACCGCTAC
	gapRTR	ACCTACAGCTTTAGCAGCAC

* For generating the fragments for DNA sepharose, forward primers (−F) were biotinated at the 5′ end.

### Purification of a DNA Binding Protein from *L. helveticus* CM4

*L. helveticus* CM4 (European Patent EP1016709) was grown at 37°C in 1 L of MRS broth [18]. Cells were harvested by centrifugation at 3,500 rpm for 10 min, and washed with 10 ml of 0.01 M Tris-HCl (pH 7.9), 0.1 M NaCl, 25% (W/V) sucrose. Then 5 ml of 0.3 M Tris-HCl (pH 7.9), 0.1 M EDTA containing 4 mg/ml lysozyme and 1 mg/ml muramidase was added and the sample was incubated at 30°C for 60 min. Cells were disrupted by adding 25 ml of 1.0 M NaCl, 0.02 M EDTA, 0.08% deoxycholate, pH 7.0. After incubating on ice for 10 min, the suspension was centrifuged at 10,000 rpm for 10 min and the supernatant was collected. The supernatant was mixed with 70 ml of 17% (W/V) polyethylene glycol 6000 (PEG), and 0.157 M NaCl containing 2% casamino acids to precipitate the chromosomal DNA for the collection of DNA-associated proteins. The mixture was centrifuged for 10 min at 10,000 rpm and the precipitated DNA-protein complex was collected and suspended in 10 ml of 5% PEG, 2 M NaCl, 10 mM Tris-HCl, pH 7.9 to release DNA binding proteins from the precipitated chromosomal DNA. DNA binding proteins were isolated in the supernatant after centrifugation for 10 min at 10,000 rpm. After dialysis against 1 L of TE buffer, 10 mM Tris-HCl (pH 7.0), 1 mM EDTA, at 4°C for 20 h, the sample was loaded onto a 2 ml DNA-sepharose column in the presence of 10 mM each of BCAAs (Val, Ile, Leu). The column was washed with five column volumes of 20 mM Hepes (pH 7.9), 50 mM KCl, 0.2 mM EDTA, 0.5 mM DTT, 10% glycerol (Hepes buffer) containing 10 mM of each BCAA. Proteins of interest were expected to lose affinity for DNA in the absence of BCAAs, and were eluted by washing the column with 5 bed volumes of Hepes buffer without BCAAs. Protein purity was analyzed by sodium dodecyl sulfate-polyacrylamide gel electrophoresis (SDS-PAGE) according to the method of Laemmli [19] using a 5–15% linear gradient polyacrylamide gel. Proteins were stained with a silver stain kit (Wako Pure Chemical Industries Ltd.).

### Protein Identification

To identify proteins, each band excised from the SDS-PAGE gel was dehydrated in 100% acetonitrile until it turned opaque white. The extracts from the bands were then dried in a vacuum centrifuge and subsequently rehydrated in 10 µl of digestion solution consisting of 50 mM CaCl_2_, 0.01 µg/µl modified sequence-grade trypsin (Promega). After incubation at 37°C for 16 h, peptides from each band were applied to MS and MS/MS spectra analyses using a MALDI-TOF/TOF (Ultraflex, Bruker Daltonics). Proteins were identified by MS/MS ion searching using MASCOT software (Matrix Science) with the NCBI nr database.

### Purification of the 26 kDa CBS Domain Protein (BCARR) from *E. coli*

**Figure 1:**
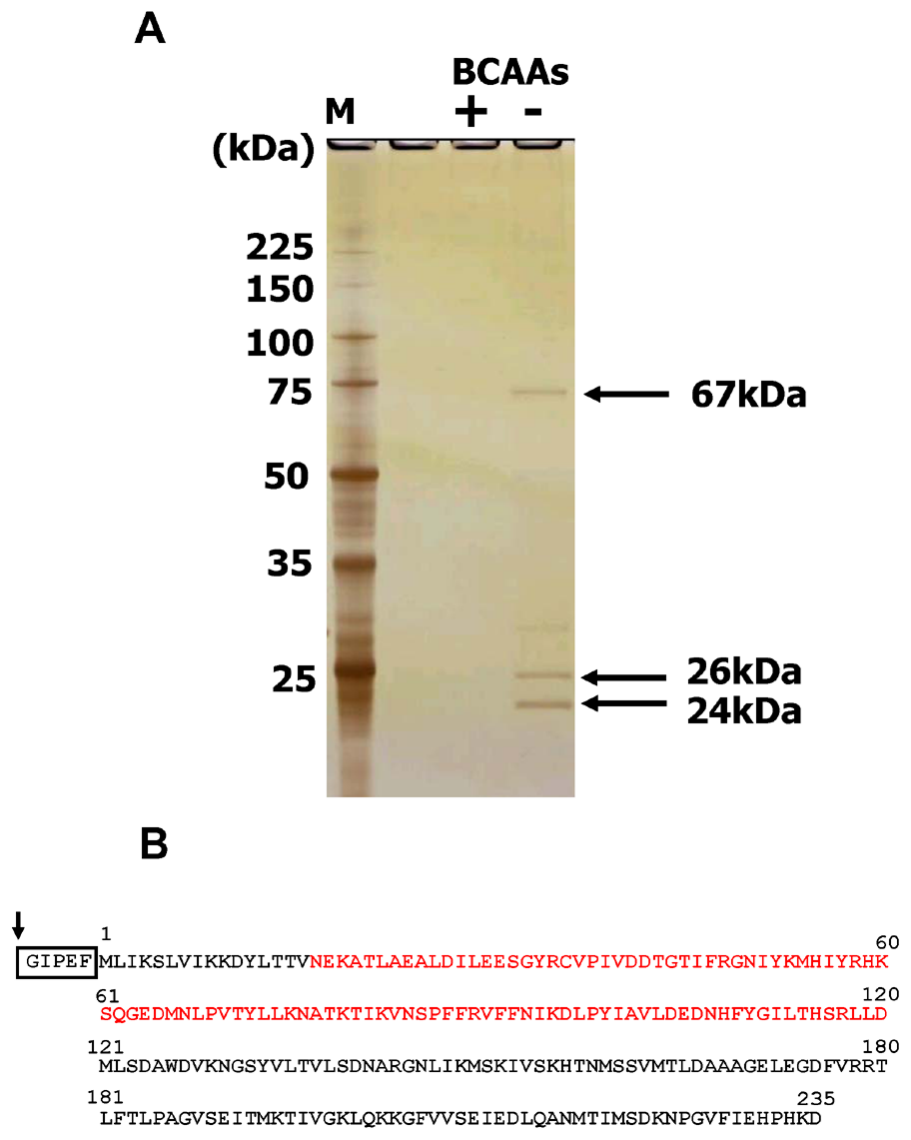
Affinity purified proteins from *L. helveticus* CM4 eluted from DNA-sepharose. DNA binding proteins purified by DNA-sepharose, which was bound to an approximately 300 bp DNA fragment corresponding to the upstream region of 6 proteolytic genes, were analyzed by SDS (5–15%)-PAGE as described in Materials and Methods (A). Proteins were eluted with buffer containing 10 mM BCAAs (Val, Leu, Ile) (+) or no BCAAs (−). The gel was silver stained after electrophoresis. The molecular masses of the marker proteins (lane M) are given on the left. Schematic drawing of the glutathione S-transferase (GST) fused 26 kDa cystathionine ß-synthase (CBS) domain protein is shown (B). The C-terminal GST protein is shown in the box. The arrow indicates the cleavage site for Factor Xa. Red letters indicate two tandem CBS domains (CBS pair) in the 26 kDa protein.

General procedures for DNA manipulation were carried out essentially as described [20]. Chromosomal DNA was purified from *L. helveticus* CM4 according to the method of Leenhouts *et al.* [21]. To prepare the glutathione S-transferase (GST) fusion protein, the gene encoding the BCARR (Branched Chain Amino acids Responsive transcriptional Regulator (DDBJ accession number: AB812553) protein was amplified with primers 26kF and 26kR (Table 1), and inserted into the *Eco*RI and *Xho*I sites of the vector pGEX 5X-1 (GE Healthcare). The resulting plasmid was introduced into *Escherichia coli* HB101 and an isolated transformant was used for purification of the GST-fusion protein. The BCARR protein was fused with GST at the C-terminus of GST (GST-BCARR protein). The GST-BCARR protein was purified with Glutathione-sepharose and digested with Factor Xa according to the manufacturer’s instructions (GE Healthcare) to obtain the BCARR protein (Figure 1B). The purity of the BCARR protein was analyzed by 5–15% SDS-PAGE as described above. The amount of protein was determined with a Protein Assay Kit (Bio-Rad Laboratories) using BSA as the standard protein.

### Quantification of *pepV* Gene Expression in the *E. coli* Transformant

To generate expression plasmids for *pepV* and the 26 kDa CBS protein (BCARR) gene, PCR products, which contained approximately 500 bp upstream and 300 bp downstream of each gene, were produced with the primers pepVexF and pepVexR for *pepV,* and 26kexF and 26kexR for the 26 kDa CBS domain protein (BCARR) gene (Table 1). The PCR products were inserted into the *Eco*RI/*Bam*HI and *Bam*HI/*Sph*I sites, respectively, in pBR322 (Takara Bio Inc) to obtain pBR-pepV and pBR-pepV-CBS. Each vector was introduced into *E. coli* HB101. To quantify the transcriptional level of the *pepV* gene of *L. helveticus* CM4 in the transformed *E. coli* HB101, reverse transcription and quantitative PCR were carried out with a Light Cycler 480 (Roche Diagnostics) and One Step SYBR PrimeScript RT-PCR Kit II (Takara Bio Inc) according to the manufacturer’s protocol. Primers for the *pepV* gene (pepVRTF, pepVRTR) and the internal reference, glyceraldehyde 3-phosphate dehydrogenase (*GAPDH*) gene (gapRTF, gapRTR) were used. To understand repressed gene expression of the proteolytic system in milk medium [11], *E. coli* transformants were cultured in M9 medium [20] with or without both 0.4% casamino acids (hydrolysate of milk casein: BD) and 10 mM BCAAs. The cells were cultured in M9 and M9+ BCAAs medium and harvested at optical densities (OD_600_) of 0.6 and 0.9, one hour after each inoculation, respectively. Total RNA was extracted from the cells with the RNeasy mini kit (Qiagen) according to the manufacturer’s protocol. The transcription levels were quantified according to the relative quantification method reported previously [22] using presumed real-time amplification efficiencies = 2. Values relative to the transcription levels of transformants harboring pBR-pepV in each medium are reported.

### In Vitro DNA Binding Assays

Electrophoresis mobility shift assays (EMSAs) were carried out according to the previously described method for the CodY protein [14] with some modifications. In short, purified BCARR (CBS domain protein) was mixed with DNA fragments of approximately 300 bp that correspond to the regions upstream of the six proteolysis genes. Binding reactions were carried out in 10 µl of reaction mixture containing 20 mM Tris-HCl (pH 8.5), 10 mM MgCl_2_, 100 mM KCl, 1 mM dithiothreitol, 15 ng of PCR fragment, 0.01% bovine serum albumin, 0 to 3 µM of BCARR protein and 10 mM of BCAAs or 10 mM each Val, Leu, Ile, Gly, His, Ser, Thr, Pro and Met. After incubation at 30°C for 10 min, protein-DNA complexes were analyzed on 5% polyacrylamide gels run in TBE buffer at 120 V for 0.5 h, followed by staining with ethidium bromide.

### Search for the BCARR Binding DNA Region Upstream of the *pepV* Gene

To identify the preferred DNA binding sequence upstream of the *pepV* gene, four short DNA fragments corresponding to the region upstream of the *pepV* gene were amplified with the four sets of primers listed in Table 1. After the PCR amplification, each fragment was labeled with [γ-^32^P]-ATP by using T4 DNA kinase. Binding reactions were carried out in 10 µl of reaction mixture containing 20 mM Tris-HCl (pH 8.5), 10 mM MgCl_2_, 100 mM KCl, 1 mM dithiothreitol, about 2,500 cpm of ^32^P labeled PCR fragment (about 0.3 ng), 0.01% bovine serum albumin, and 2.3 µM of BCARR protein and with 10 mM BCAAs. Protein-DNA complexes were analyzed by 10% polyacrylamide gel electrophoresis as described above, and ^32^P signals were detected by exposure to X-ray film, RX (Fuji Film).

### DNase I Footprinting Analysis using Purified BCARR Protein

DNase I footprinting analysis using purified BCARR protein was performed according to the method described previously [23]. After the PCR amplification of a 309 bp DNA fragment from upstream of the *pepV* gene using 5′-OH primers, the DNA fragment was labelled with [γ^−32^P]-ATP by using T4 DNA kinase. Then, the radioisotope-labeled DNA fragments were cut with *Alu*I to generate a DNA fragment labeled with ^32^P at one end (−266 to +24). Binding reactions were carried out in 10 µl of reaction mixture containing 20 mM Tris-HCl (pH 8.5), 10 mM MgCl_2_, 100 mM KCl, 1 mM dithiothreitol, about 3 ng of ^32^P labeled PCR fragment (about 100,000 cpm), and 0.01% bovine serum albumin with 0, 2.3 and 18 µM BCARR protein. After incubation at 30°C for 10 min, protein-DNA complexes were treated with 0.125 units of DNase I for 1.5 min at 37°C. After heating at 90°C for 1 min, the DNase I treated samples were analyzed by electrophoresis on a 5% polyacrylamide gel containing 8 M urea in TBE buffer, and ^32^P signals were detected by exposure to X-ray film (Fuji Film). An A+G sequence ladder was prepared by the method of Maxam and Gilbert [24].

### Phylogenetic Analysis of the BCARR

Homologous protein sequences were identified by using BLASTP (cutoff e-value >10^−50^). An unrooted phylogenetic tree with branch lengths displayed was built using ClustalW and the NJ algorithm. To simplify the tree, one sequence was selected from each genus. For the genus *Lactobacillus*, four sequences were selected from the delbruekii subgroup including *L. helveticus*, and one sequence from each subgroup other than the delbruekii subgroup as defined in the previous report [25].

## Results

### Purification and Identification of DNA Binding Proteins

To collect DNA binding proteins, intracellular components with affinity to *L. helveticus* CM4 genomic DNA were co-precipitated with chromosomal DNA by adding polyethylene glycol 6000 as described in Materials and Methods. The precipitated proteins released from DNA by adding 1 M NaCl were then applied to an affinity column in the presence of BCAAs. The affinity column contained DNA-sepharose bound PCR fragments corresponding to the DNA upstream of the *pepV, pepO, pepO2, pepT2, pepCE* and *dppD* genes. Proteins with molecular masses of 24, 26 and 67 kDa were specifically released from the affinity resin by washing with BCAA-free Hepes buffer (Figure 1A). The three proteins were identified by sequence analyses of peptides released from each protein band. The 67 kDa protein was identified as a prolyl-tRNA synthase, which would not be expected to bind to DNA. In contrast, the 26 kDa and 24 kDa proteins were identified as same protein with homology to the 26.3 kDa protein lhv_0288 (YP_001576806.1) of *L. helveticus* DPC 4571 (NC_010080.1). This result indicated that the 24 kDa protein might be a degradation product of the 26 kDa protein. The 26 kDa protein has two tandem cystathionine β-synthase domains (CBS pair) at the amino terminus as reported previously [26] (Figure 1B). CBS catalyzes the formation of cystathionine from homocysteine and serine. CBS domains with no catalytic domain [26] are widely distributed in most species of life but their functions are largely unknown. A recent study suggested that the CBS domain protein MJ0729 from *Methanocaldococcus jannaschii* might bind to the E-box of DNA [27]. These results suggested that the 26 kDa protein with a CBS domain (CBS domain protein) might have affinity for DNA upstream of the *pepV*, *pepO*, *pepO2*, *pepT2*, *pepCE* and/or *dppD* genes in the presence of BCAAs.

### DNA Binding of the CBS Domain Protein

**Figure 2:**
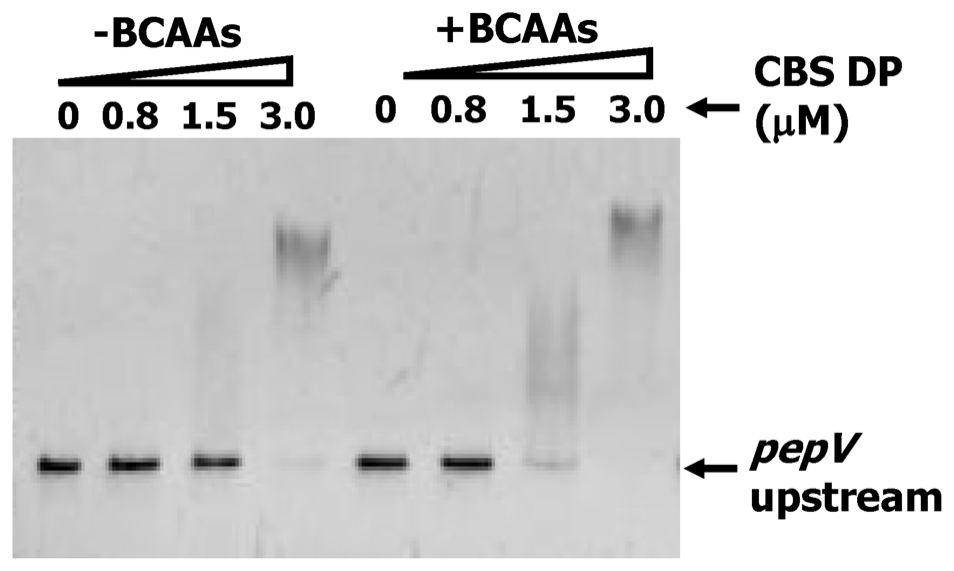
Electrophoresis mobility shift assay showing DNA binding. Electrophoresis mobility shift assay using the purified CBS domain protein (CBSDP) and a 309 bp DNA fragment from upstream of the *pepV* gene in the presence or absence of 10 mM BCAAs and various amounts of CBS domain protein.

To confirm the DNA binding ability of the 26 kDa CBS domain protein from strain CM4, the corresponding gene was amplified by PCR using primers 26 kF and 26 kR (Table 1) to prepare a glutathione S-transferase (GST) fusion protein by the method described in Materials and Methods. The GST fusion protein was expressed in *E. coli* HB101, and the CBS domain protein was purified by using a glutathione-sepharose affinity column (Figure 1B). Then, electrophoresis mobility shift assays (EMSAs) were carried out with the purified protein and a 309 bp DNA fragment corresponding to the *pepV* promoter region in the presence or absence of 10 mM BCAAs. The DNA band was shifted by 3 µM CBS domain protein in the absence or presence of BCAAs (Figure 2). However, a band shift was observed with 1.5 µM CBS domain protein only when BCAAs were present (Figure 2).

To understand the effect of various single amino acids on the binding of the CBS domain protein to DNA, 10 mM each of Val, Leu, Ile, Gly, His, Ser, Thr, Pro or Met was used in EMSAs with the *pepV* upstream probe. Each BCAA promoted CBS domain protein binding to the *pepV* gene, and Ile was most effective. However, no shift was observed in EMSAs with the other tested amino acids (Gly, His, Ser, Thr, Pro or Met) (data not shown). These results also suggest that Ile has the highest affinity for the ACT domain, and the BCARR-Ile complex is capable of binding to the target DNA. Unlike *Bacillus subtilis* CodY, the transcriptional regulator that senses BCAAs in *B. subtilis*, GTP was not necessary for the CBS domain protein to bind to DNA (data not shown).

### Transcriptional Regulation of the CBS Domain Protein (BCARR)

**Figure 3:**
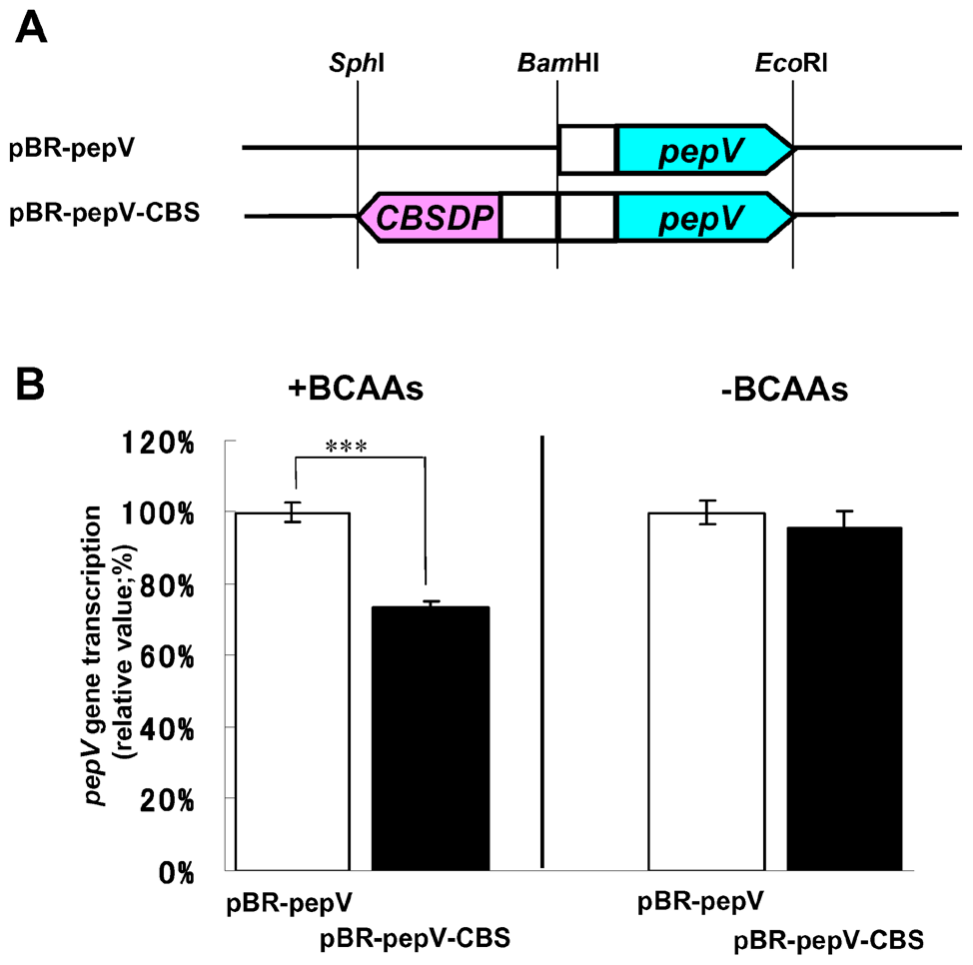
Transcriptional regulation by the CBS domain protein. Schematic drawing of the expression plasmid (A). The *pepV* gene including 500 bp of upstream sequence was expressed in *E. coli* HB101 cells carrying pBR-pepV. The gene encoding the CBS domain protein was also co-expressed with the *pepV* gene in *E. coli* HB101 harboring pBR*-*pepV-CBS. White boxes show about 500 bp of DNA upstream of the genes. *CBSDP* and *pepV* indicate the ORF of each gene. *pepV* gene transcription in the *E. coli* transformant was quantified by RT-PCR with total RNA from *E. coli* cultured with or without 0.4% casamino acids and 10 mM BCAAs (B). The glyceraldehyde 3-phosphate dehydrogenase (*GAPDH*) gene (gapRTF, gapRTR) was used as the internal reference. *Error bars* indicate standard deviations. Statistical analysis of the data from triplicate experiments was conducted using the Student’s *t* test. ***P<0.001.

*L. helveticus* CM4 is a non-transformable strain and therefore we were unable to isolate a mutant strain lacking the gene encoding the CBS domain protein. Therefore, to understand the influence of the CBS domain protein on the transcriptional level of the proteolysis system of *L. helveticus* CM4, the *pepV* gene was expressed in *E. coli* HB101 cells with or without co-expression of the gene encoding the CBS domain protein. The *pepV* gene was expressed in *E. coli* HB101 by introduction of pBR-pepV, which includes the *pepV* ORF and about 500 bp of upstream DNA. Then, the CBS domain protein gene was co-expressed with the *pepV* gene in *E. coli* HB101 harboring pBR-pepV-CBS (Figure 3A). The transcription levels of the *pepV* gene in both strains, quantified by real-time PCR, were not changed in M9 minimal medium (Figure 3). On the other hand, *pepV* gene transcription was decreased to 73% in *E. coli* harboring pBR-pepV-CBS compared to pBR-pepV, when casamino acids and BCAAs were present in the medium. These results suggest the possibility that the CBS domain protein expressed in *E. coli* HB101 binds upstream of the *pepV* gene in response to BCAAs and represses transcription of the *pepV* gene. The CBS domain protein may be a novel type of regulatory protein involved in controlling the transcription of the proteolytic system by sensing BCAAs in *L. helveticus*. Therefore, the CBS domain protein was named Branched Chain Amino acids Responsive Transcriptional Regulator (BCARR), and the sequence was submitted to DDBJ (accession number: AB812553).

### Consensus DNA Motif Search

**Figure 4:**
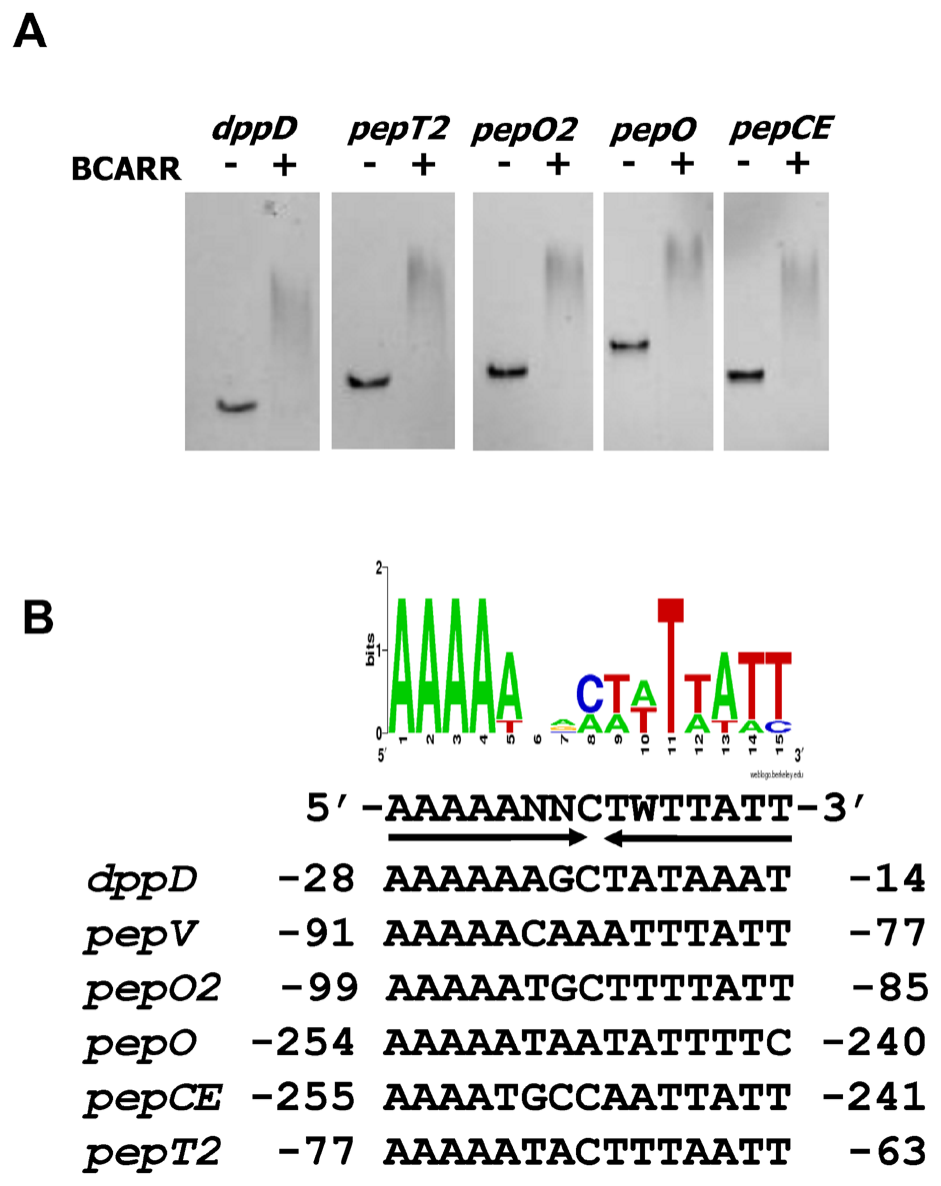
The search for a consensus motif for BCARR binding. Electrophoresis mobility shift assays with the promoter regions of proteolysis genes using the purified BCARR protein (A). The upstream regions of the *pepT2, pepCE, pepO, pepO2* and *dppD* genes were incubated alone or with 2.2 µM BCARR protein and 10 mM BCAAs. A conserved motif was identified for these six upstream sequences by MEME analysis [28] (B). The weight matrix shows the frequency of A, C, T, or G nucleotides (as indicated in the legend) at each position of the motif. The inversely repeated consensus sequence (indicated by arrows) deduced from these frequencies is shown below the diagram. W can be either A or T. N can be any nucleotide. A graphical representation of the identified motif was obtained at the Weblogo website (http://weblogo.berkeley.edu/logo.cgi). Nucleotide positions are relative to the start codon of each proteolysis gene or the first gene in each operon (*pepCE* and *pepO*).

To evaluate the binding of the BCARR protein to specific DNA fragments containing the promoters of the *pepO, pepO2, pepT2, pepCE* and *dppD* genes, which are negatively regulated in response to added peptides [11], EMSAs were carried out in the presence of BCAAs. The results demonstrated that DNA fragments from upstream of the *pepO*, *pepO2*, *pepT2*, *pepCE* and *dppD* genes were shifted when the BCARR protein and BCAAs were present in the reaction mixture (Figure 4A). A consensus DNA motif for binding of the BCARR protein was predicted in the regions upstream of the *pepV*, *pepO*, *pepO2*, *pepT2*, *pepCE* and *dppD* genes, by using MEME analysis [28]. An AT-rich sequence containing the palindromic DNA sequence 5′-AAAAANNCTWTTATT- 3′, which was present upstream of each gene ranging from −255 to −14 from the start codon, was predicted to be the BCARR binding motif (BCARR-box) (Figure 4B). Moreover, the consensus sequence motif was observed upstream of other genes involved in amino acid metabolism and transport, which were shown to be down-regulated in response to added peptides in a previous study [11], such as the *hisM* operon (position; -59/−45 from start codon), the *potE* gene (−181/−167), the *lysA* operon (−54/−40), and the *serC* operon (−21/−6).

### Selected Binding of BCARR to the Region Upstream of the *pepV* Gene

**Figure 5:**
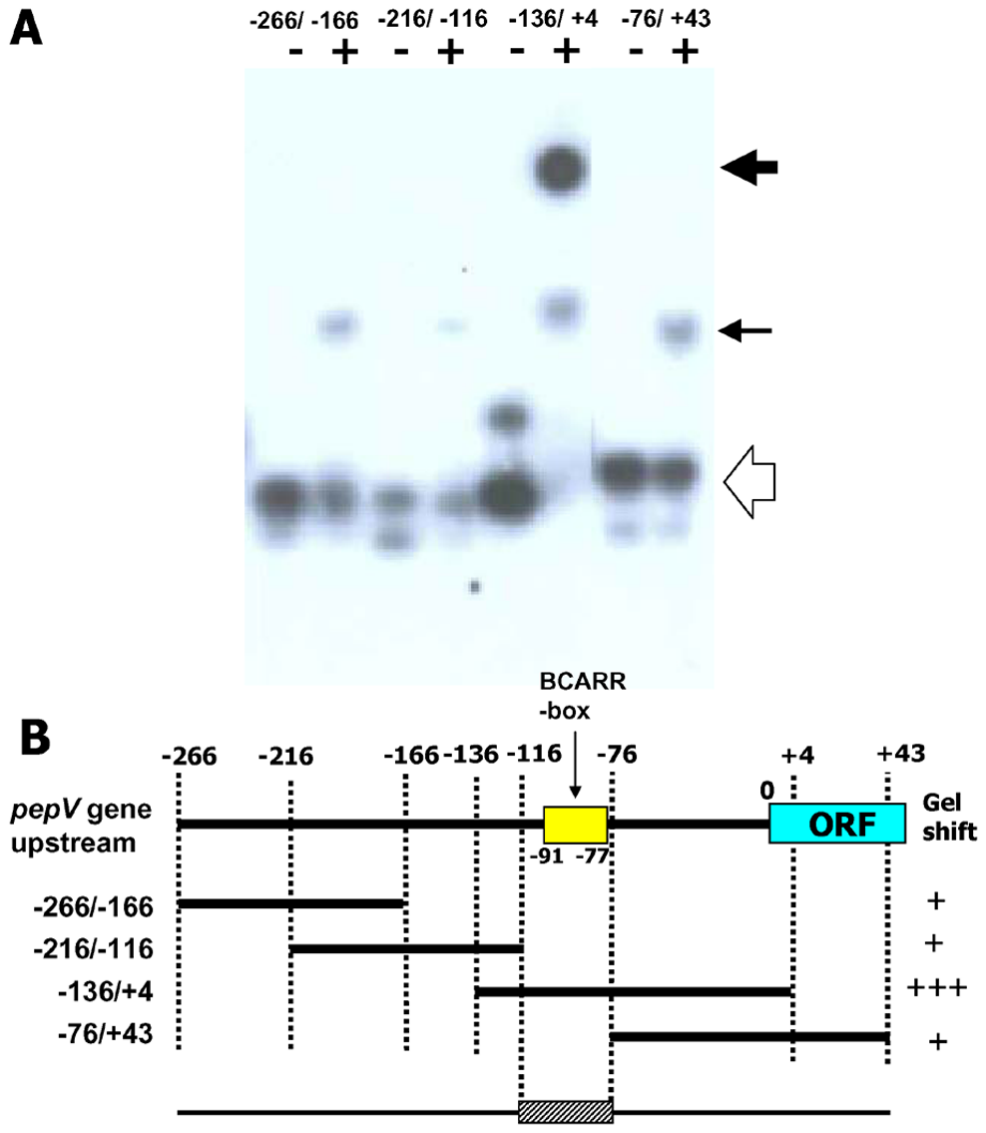
Electrophoresis mobility shift assay with various DNA fragments of the region upstream of the *pepV*. Mobility shift assay with or without 2.3 µM BCARR protein and 10 mM BCAAs (A). Each 2,500 cpm of ^32^P labeled DNA fragment (0.3 ng) was incubated as described in Materials and Methods. Protein-DNA complexes were analyzed by 10% polyacrylamide gel electrophoresis. The ^32^P signals in the gel were detected by exposure to X-ray film. The position of the shifted bands is indicated in the right margin by arrows (weak shift indicated by a small arrow, and a significant shift by a big arrow; the origin is indicated by the open arrow). DNA fragments used in the above experiment and the results of the EMSA are summarized in (B). Nucleotide positions are relative to the start codon. BCARR-Box indicates the predicted BCARR binding motif by MEME analysis. ORF shows the open reading frame of the *pepV* gene. The intensity of the shifted bands is indicated by + (slight probe shifted) to +++ (all probes shifted).

To examine whether the BCARR protein preferably binds to the specific DNA sequence predicted as the BCARR-box (Figure 4B), EMSAs were carried out using various DNA fragments corresponding to the region upstream of the *pepV* gene as illustrated in Figure 5B. As shown in Figure 5A, DNA fragments for DNA_−266/−166_, DNA_−216/−116_, DNA _−136/+4_ and DNA _−76/+43_ were slightly shifted when the BCARR protein was added in the presence of BCAAs (Figure 5A). As expected, DNA _−136/+4_, which contains the predicted BCARR-box was highly shifted when 3 µM BCARR protein and 10 mM BCAA were added (Figure 5B). The above observation reveals that a DNA fragment from −116 to −76 predicted by comparative sequence analysis, which contains the BCARR-box (−91 to −77), might bind with the highest affinity to BCARR in the presence of BCAAs as illustrated in Figure 5B.

### Footprint Analysis of the *pepV* Gene with the Purified BCARR Protein

**Figure 6:**
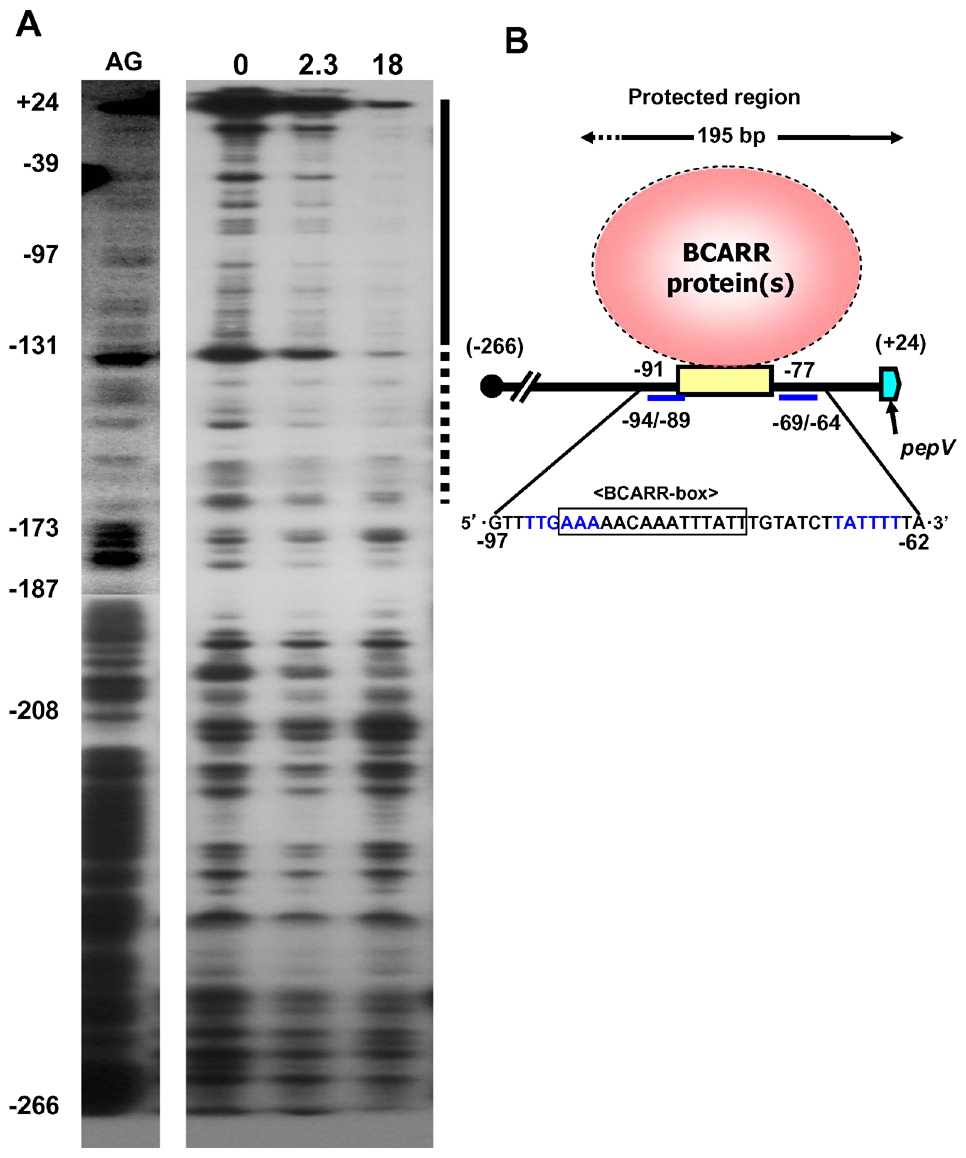
DNase I footprinting analysis and schematic drawing of the BCARR protein-DNA binding. Single-end (forward 5′strand) radioactively labeled probes containing 290 bp of sequence upstream of *pepV* were examined alone (lane 1) or with 2.3 µM (lane 2) and with 18 µM (lane 3) BCARR protein (A). Lane AG; A+G ladder prepared from the labelled DNA as described in Materials and Methods. The numbers at the left indicate the nucleotide number from the start codon. The vertical bar at the right represents the protected region. Solid lines and dotted lines indicate strongly and weakly protected regions, respectively. Schematic drawing of the DNaseI footprint analysis (B). The binding site of the BCARR protein was located in the sequence upstream of the *pepV* gene. The consensus motif detected in the upstream sequences of the six proteolysis genes ranging from −91 to −77 in *pepV* gene is shown by a yellow box. The putative promoters, −35 (TTGAAA) and −10 (TATTTT), are underlined and in blue letters. The nucleotide numbers indicate the distance from the start codon.

To examine whether the BCARR protein binds the predicted DNA sequence (BCARR-box) in the *pepV* promoter region, DNase I footprinting analysis was performed. A 290 bp long DNA fragment carrying the promoter region of the *pepV* gene (−266 to +25), which was radioactively labeled at the 5′ end of the forward strand, was used. No protection was observed if no BCARR protein was added to the reaction mixture (Figure 6A). However, the BCARR protein protected approximately 195 bp of the *pepV* promoter region when 18 µM BCARR protein was present in the reaction mixture (Figure 6A, lane 3). These results demonstrate that the BCARR protein interacts with an approximately 195 bp long region of the *pepV* promoter, protecting the BCARR-box from −91 to −77 and the −35 and −10 promoter sequences as illustrated in Figure 6B. These results also strongly suggest that the BCARR protein might affect the transcription of the *pepV* gene by binding and covering the promoter region (−35 and −10).

### Prediction of the BCAA Sensing Domain Structure in the BCARR Protein

**Figure 7:**
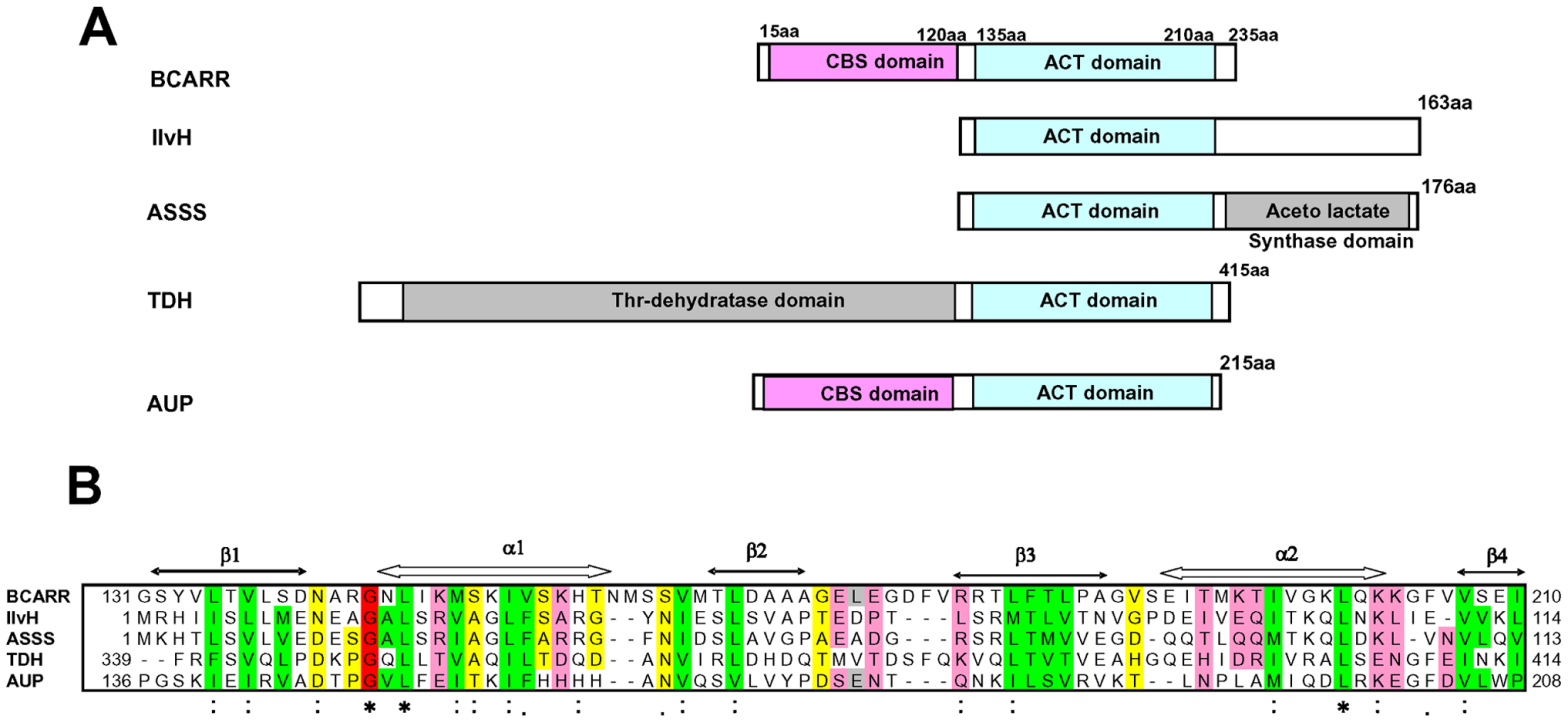
Homology search and sequence alignment of the ACT domain of the *L. helveticus* BCARR protein. Predicted schematic domain structure of the *L. helveticus* BCARR protein and proteins with similarity to the C-terminal region of the BCARR identified by PSI-BLAST (A). Sequence alignment of the ACT domain of the *L. helveticus* BCARR protein and reported ACT domain proteins (B). The sequence alignment was performed using ClustalW. Helices and beta-strands are represented by arrows and are labeled “α” and “β” respectively. The color scheme generally follows that of Grant *et al.* [23], and Aravind and Koonin [41] and refers to the following residue types: green, hydrophobic (ILVCAGMFYWTP); magenta, polar (HKREQDNST); gray, large (FILMWYKREQ); yellow, small (ACGSTDNVP); and red, conserved glycine. IlvH; Acetohydroxylate synthase small regulatory subunit [*Nitrosomonas europaea*] (ref|NC_004757.1|), ASSS; Acetolactate synthase small subunit [*Synechococcus* sp. WH 7803] (emb|CAK24070.1|), TDH; threonine dehydratase [*Selenomonas noxia* F0398] (gb|EHG23294.1|), AUP; acetoin utilization protein [*Lysinibacillus sphaericus* C3-41] (ref|YP_001699789.1|). Symbols “ * ”, “ : ” and “. ” are shown according to the method of ClustralW. ‘*’ indicates positions are completely conserved. ‘:’ indicates a fully conserved ‘strong’ group. ‘.’ indicates one of the fully conserved ‘weaker’ groups.

To understand the structural features of the BCARR protein, a possible domain motif for sensing amino acids at the C-terminal region of the BCARR protein was predicted (131–235 aa) by PSI-BLAST homology searching. An ACT domain composed of four β strands and two α helices arranged as a βαββαβ fold was observed at the C-terminus of BCARR (Figure 7). ACT domains, named after the first letters of three of the proteins aspartate kinase-chorismate mutase-tyrA (prephenate dehydrogenase), have been reported to have the ability to bind amino acids and function to regulate certain aspects of amino acid metabolism. Based on the alignment analysis, the glycine residue is most likely important in the binding pocket as it is well conserved in the ACT domain of BCARR (Figure 7). These findings suggest that the 26 kDa BCARR protein might sense BCAAs at the C-terminal region and the association might increase its affinity for DNA when BCAAs are present.

### Phylogenetic Analysis of *L. helveticus* CM4 BCARR Protein

**Figure 8:**
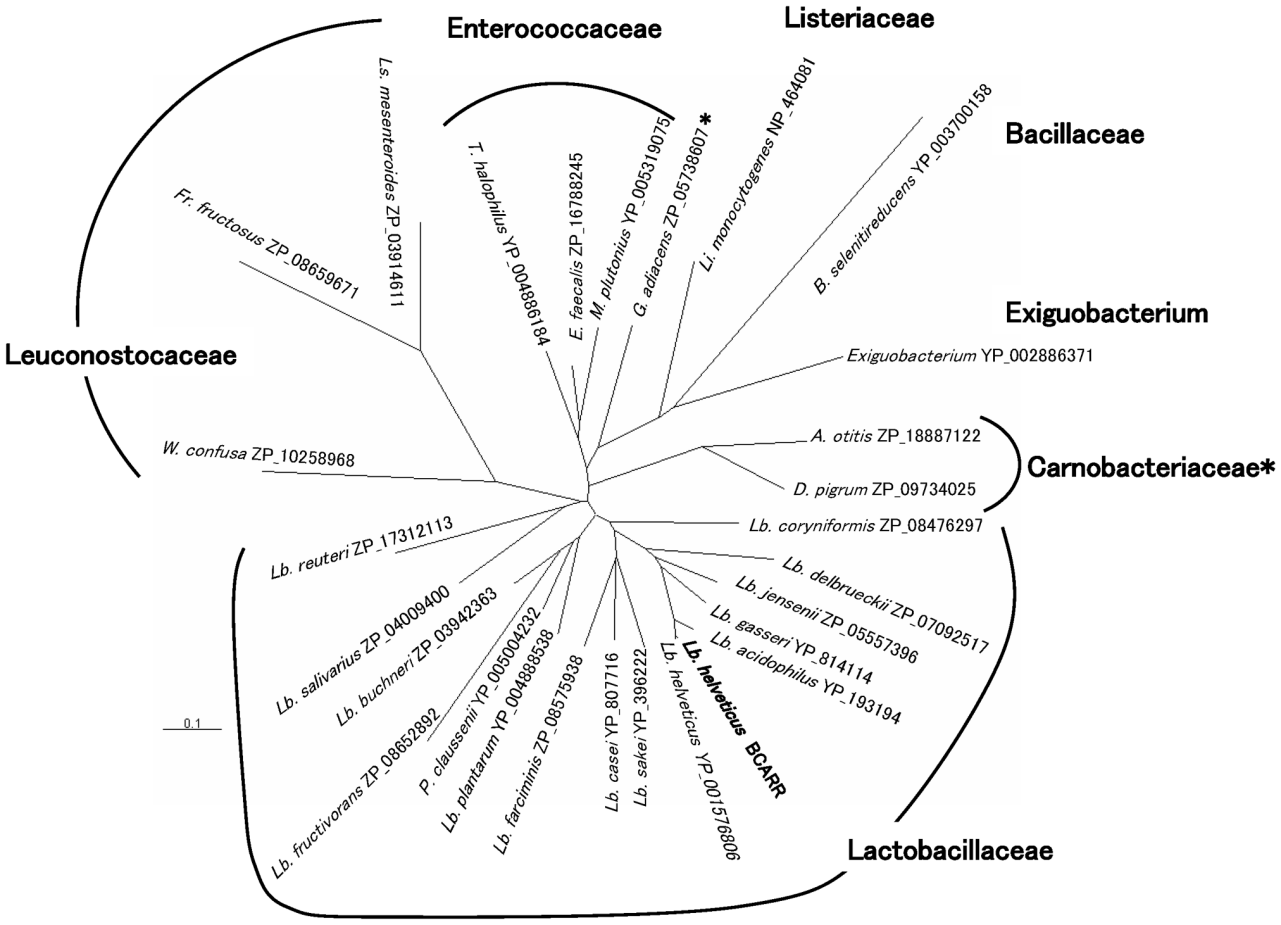
The phylogenetic tree of the BCARR. Homologous protein sequences were identified by BLASTP searches (cutoff e-value >10^−50^). An unrooted phylogenetic tree displaying branch lengths that was built using ClustalW and the NJ algorithm is shown. To simplify the tree, one sequence was selected from each genus. For the genus *Lactobacillus*, four sequences were selected from the *delbruekii* subgroup including *L. helveticus*, one sequence from each subgroup other than the *delbruekii* subgroup as defined in the previous report [25]. The scale bar represents base changes per site. *A. : Alloiococcus, B. : Bacillus, D. : Dolosigranulum, E. : Enterococcus, F. : Fructobacillus, G. : Granulicatella, Ls. : Leuconostoc, Li. : Listeria, M. : Melissococcus, P. : Pediococcus, T. : Tetragenococcus, W. : Weissella.* * Carnobacteriaceae also includes *G. adiacens*.

Phylogenetic analysis of the *L. helveticus* CM4 BCARR protein revealed the presence of homologs in lactobacillaceae, enterococcaceae, leuconostocaceae, carnobacteriaceae, listeriaceae, exiguobacteria and bacillaceae (Figure 8).

## Discussion

In our previous study, transcriptional down-regulation of the proteolytic system and reduced release of the antihypertensive peptides VPP and IPP were observed in *L. helveticus* CM4 fermented milk when peptides were added into the fermented milk [11]. In the present study, we successfully identified a 26 kDa CBS domain protein by affinity purification with DNA from regions upstream of proteolysis genes that were repressed in response to peptides [11]. The repression of *pepV* gene expression in the *E. coli* transformant expressing the CBS domain protein gene compared to the control strain without the CBS domain protein gene was not large (73%), but was similar to the repression by 2% peptides in *L. helveticus* CM4 [11]. Here we named this novel 26 kDa CBS domain protein, which functions to down-regulate proteolysis gene expression in response to BCAAs in *L. helveticus*, BCARR (Branched Chain Amino acids Responsive Transcriptional Regulator). This protein may function as a negative transcriptional regulator for the proteolytic system that allows cells to conserve energy when sufficient amino acids are present. *cis* and *trans* elements of the CodY regulon, which regulate proteolysis genes in response to intracellular amino acids, have been reported in *L. lactis* [14], *B. subtilis* [29], *Streptococcus thermophilus* [30], *Streptococcus pneumoniae* [31], *Streptococcus mutans* [32] and *Oenococcus oeni* [33], but not in lactobacilli. Phylogenetic analysis of the *L. helveticus* CM4 BCARR protein revealed the existence of homologs in lactobacillaceae, enterococcaceae, leuconostocaceae, carnobacteriaceae, listeriaceae, exiguobacteria and bacillaceae (Figure 8). Among them, enterococcaceae, listeriaceae and bacillaceae (one species; *Bacillus selenitireducens*) have homologs of both CodY and BCARR based on BLASTP searches (cutoff e-value >10^−50^) as described in Materials and Methods. On the other hand, no BCARR homologs were present in the streptococcaceae, including lactococci, and bacillaceae, which have a CodY homolog that regulates the proteolysis system. The roles of the two types of transcriptional regulators in the enterococcaceae, listeriaceae and bacillaceae (one species; *Bacillus selenitireducens*) are still not clear. To understand the role of each protein, isolation and characterization of knock-out strains will be needed.

Cystathionine β-synthase (CBS) catalyzes the formation of cystathionine from homocysteine and serine. CBS has been conserved in eukaryotic evolution and is involved in the removal of homocysteine from the methionine cycle. In humans, a CBS deficiency results in an elevated level of circulating homocysteine (homocystinuria), which is a risk factor for a number of neurological defects and vascular diseases. However, the presence of a CBS domain motif with no catalytic domain has been reported in various proteins [26] as observed at the N-terminal region of BCARR in the present study. CBS domains are widely distributed in most species of life but their functions are largely unknown. Although their functions are unknown, a previous study suggested that an archaeal CBS domain protein binds to DNA in *Methanocaldococcus jannaschii* [27]. Relatively little is known about the role of CBS domain proteins as transcriptional regulators in bacteria and until now there have been no reports of CBS proteins binding to specific regulatory sequences in response to BCAAs. Interestingly, an ACT domain containing a βαββαβ-motif, which is thought to be a common regulatory structure in amino acid metabolic enzymes and transcriptional regulators [23], [34], was predicted at the C-terminal region of the BCARR from 135 aa to 210 aa (Figure 7). The majority of proteins containing ACT domains appear to interact with amino acids and be involved in some aspect of regulation of amino acid metabolism [35], [36]. The presented results suggest that BCAAs bind to the ACT domain at the C-terminus of the BCARR protein and the complex increases the affinity of the CBS domain binding to a DNA sequence motif upstream of proteolysis genes (BCARR-box) as shown by the footprint analysis (Figure 6), thereby preventing RNA polymerase from binding to the promoters and repressing the transcription of the downstream genes. The reported results also support the down-regulation of other proteolysis genes listed in Figure 4, each with an upstream BCARR-box. The positions of the BCARR boxes upstream of the *pepO* and *pepCE* genes were relatively far from the ORFs; however, the wide area of DNA protection by BCARR could repress the transcription of these genes.

The search for a common DNA motif in the promoter regions of the six genes resulted in the identification of a unique AT-rich sequence containing a palindromic DNA sequence, 5′-AAAAANNCTWTTATT- 3′ (Figure 4B). The consensus motif was observed upstream of other *L. helveticus* genes that are down-regulated in response to added peptides, as observed in the previous study [11], such as the *hisM* operon, the *potE* gene, the *lysA* operon, and the *serC* operon. Furthermore, EMSAs for various DNA fragments corresponding to the region upstream of the *pepV* gene revealed that the predicted consensus motif of the *pepV* gene (BCARR-box) from −91 to −77 bp from the start codon was contained in the sequence (−116 to −76) that bound with the highest affinity to the BCARR protein (Figure 5). Footprint analysis also demonstrated binding of the BCARR protein to the *pepV* promoter region (Figure 6). However, there was no similarity of the predicted BCARR protein binding DNA motif to the codY-box DNA motif (5′-AATTTTCWGAAAATT-3′) reported in *L. lactis* [14]. Moreover, no highly conserved CodY helix-turn-helix (HTH) motif (AS++AD++GITRSVIVNALR) [37] was found in the BCARR protein. With respect to the similarity between the BCARR protein of *L. helveticus* CM4 and CodY in *L. lactis* [14], the most significant band shifts depended on Ile and BCAAs levels, and the BCAA level needed for effective EMSA band shifts (2.5 mM for the BCARR protein and 5 mM for CodY [38]). Moreover, neither protein needs GTP, which is an indicator of the energy state of the cells. A different but AT-rich sequence was also required for DNA binding of both the BCARR protein in *L. helveticus* (Figure 4B) and CodY in *L. lactis* [38] when BCAAs were present in EMSA analyses. These similarities in BCAA sensing and binding to AT-rich DNA upstream of proteolysis genes by the nonhomologous BCARR and CodY proteins are particularly interesting.

The wide and higher band shift observed in response to BCAAs in EMSAs (Figure 2, 4A, 5A) suggests the possibility of oligomerization of the BCARR protein in the DNA-protein complex. Oligomerization of proteins containing CBS domains was suggested in a previous study [39]. The oligomerization of the BCARR protein may be triggered after the formation of the BCARR-DNA complex because higher band shifts were observed when a short DNA fragment containing the BCARR-box was used (Figure 5) and large BCARR-DNA complex formed when a large amount of the BCARR protein was used in DNA footprint analysis (Figure 6).

**Figure 9:**
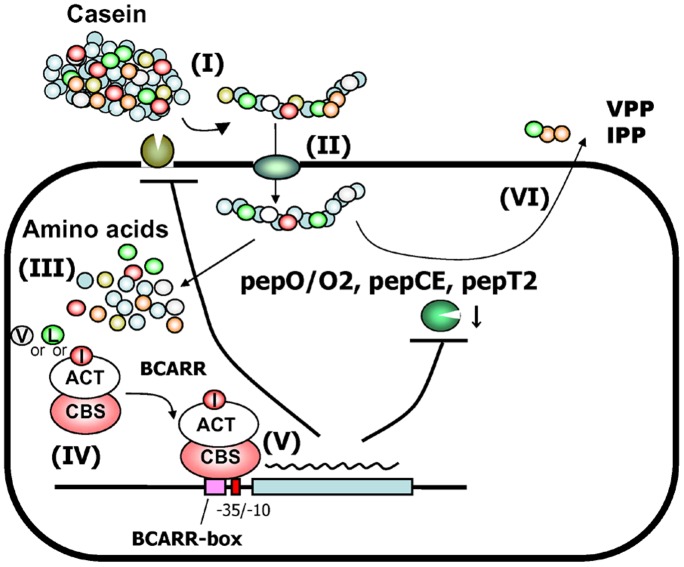
Model for the regulation of the proteolytic system of *L. helveticus* by the BCARR protein. Release of the long peptide containing VPP and IPP sequences by an extracellular proteinase (I), uptake of the long peptide via oligopeptide transporter (II), intracellular processing of the peptides to amino acids by peptidases (III), BCARR (ACT domain)-BCAA complex formation (IV), binding of the complex (CBS domain) to the BCARR-box and repression of proteolysis gene transcription (V) and repressed release of the antihypertensive peptides, VPP and IPP (VI). L; Leucine, I; Isoleucine, V; Valine. T arrows indicate repressive effects.

A mechanism for the repression of VPP and IPP release in CM4 fermented milk is presented in Figure 9. Long peptides containing VPP and IPP sequences released by an extracellular proteinase (I) from milk casein [40] enter the cell via an oligopeptide transporter (II). The long peptides are then processed by intracellular peptidases, including the endopeptidases PepO and PepO2, and tripeptidase, PepT2, and the aminopeptidase, PepCE [11]. BCAAs released from intracellular peptides may be associated with BCARR at the ACT domain (III) and the complex may increase the affinity for promoter regions of these proteolysis genes via the CBS domain region (IV), which represses expression of the proteolysis genes (V) and lowers the levels of VPP and IPP released (VI) in *L. helveticus*. This model also suggests that there would be an increased amount of bioactive peptides in a mutant strain lacking the BCARR protein.
